# Head Loss in Thin-Walled Drip Tapes with Continuous Labyrinth

**DOI:** 10.1155/2019/8640893

**Published:** 2019-12-10

**Authors:** Verônica G. M. L. Melo, Ana C. S. Araújo, Antonio P. Camargo, Leonardo L. Melo, José A. Frizzone, Wagner W. A. Bombardelli

**Affiliations:** ^1^Biosystems Engineering Department, College of Agriculture “Luiz de Queiroz” (ESALQ), University of São Paulo (USP), Piracicaba, SP, CEP 13418-900, Brazil; ^2^College of Agricultural Engineering (FEAGRI), State University of Campinas (UNICAMP), Campinas, SP, CEP 13083-875, Brazil

## Abstract

Thin-walled drip tapes with continuous labyrinth have been used for irrigation of vegetables and other short-cycle crops, especially due to their low cost. The continuous labyrinths welded into the pipe inner wall affect the head loss along such emitting pipes. In addition, the flow cross section of thin-walled pipes may change due to the effects of the operating pressure, which also has consequences for the head loss. The objective of this work was to investigate experimentally the friction factor and the head loss on thin-walled drip tapes with continuous labyrinths operated under various pressures. Two models of commercial thin-walled drip tapes with continuous labyrinths were evaluated. Nonperforated samples were used to determine the head-loss equations. The equations were adjusted as a function of flow rate and pressure head at the pipe inlet. Alternatively, the diameter in the Darcy–Weisbach equation was adjusted as a function of the pressure head by a power-law model. The possibility of using a mean diameter in the Darcy–Weisbach equation was also analyzed. Experimental investigation indicated that the friction factor in the Darcy–Weisbach equation can be accurately described using a power-law model, like the Blasius equation, but characterized by a coefficient *a*=0.3442 for the Turbo Tape and *a*=0.3225 for the Silver Tape. The obtained values of *a* are larger than those generally used and available in the literature. The influence of the operating pressure on the pipe diameter can be neglected for the purpose of calculating the head loss. The two approaches, considering the variation of the diameter with the pressure head and considering an optimum average diameter for the calculation of head loss by the Darcy–Weisbach equation, produce similar results, allowing accurate prediction of head loss. Evaluating the proposed mathematical models, 95% of predictions presented relative errors of head loss smaller than 5%. For the Turbo Tape, the optimum diameter for the purpose of calculating the head loss is 16.01 mm, which is very close to the value indicated by its manufacturer (15.9 mm). For the Silver Drip, the optimum diameter is 15.71 mm, while the manufacturer gives a value of 16.22 mm, which produces considerable error in the calculation of head loss.

## 1. Introduction

Drip irrigation is potentially the most efficient way of applying water when compared to other irrigation methods [1–3], since it minimizes water losses, saves energy, and maintains high crop production levels. The use of polyethylene collapsible emitting pipes with integrated emitters (i.e., thin-walled drip tapes) to irrigate horticultural crops has received wide attention in recent years. This is mainly due to the low cost of this material together with adequate hydraulic performance, application efficiency, and reduced installation costs of the irrigation system [3].

Recently, the industry has been disseminating another low-cost option for the irrigation of vegetables and other seasonal crops. Commercially, the material is called thin-walled drip tapes with continuous labyrinth. These emitting pipes, generally supplied in rolls, have inner diameters of approximately 16 mm, wall thicknesses of 6 mil (150 *μ*m), 8 mil (200 *μ*m), and 10 mil (250 *μ*m) and operate at pressures below 100 kPa. The geometric shape of the cross section depends on the working pressure. When not pressurized, the pipes tend to be flat and the degree of circularity increases with the working pressure. Due to the material elasticity, the diameter tends to increase to a certain extent as the working pressure is raised [4, 5]. Changes in the flow section geometry affect the flow velocity profiles inside the pipe, interfering in the friction factor and consequently in the head loss. In addition, the pressure head along the lateral is influenced by the friction head loss and changes in the terrain elevation. Along the lateral, pipe geometry may change due to decreases in pressure head and consequently the calculation of friction head loss becomes more complex [6].

The Darcy–Weisbach equation can be considered the standard method of estimating head loss, although there might be simpler ways to calculate the pressure drop in pipes [7]. The variables involved in calculating the head loss (*J*, m·m^−1^) by the Darcy–Weisbach equation (equation (1)) are the friction factor (*f*, dimensionless), which is a function of the Reynolds number (*R*_*e*_, dimensionless); the pipe internal roughness (*ε*, m); the flow rate (*Q*, m^3^·s^−1^); and the internal diameter of the pipe (*D*, m):

(1) $$\begin{array}{c} J=f\frac{1}{D}\;\frac{V^{2}}{2g}=\frac{8f}{g\pi^{2}}\;\frac{Q^{2}}{D^{5}}. \end{array}$$

The friction factor is usually determined by the Colebrook–White equation, which is an implicit combination of the Prandtl and von Karman equations. The solution is reached by iteration or by the Moody diagram. In order to facilitate the calculation of *f*, several explicit mathematical approaches have been developed and evaluated [8], and their accuracy is compared against the Colebrook–White equation.

The accuracy of the Colebrook–White equation for small-diameter plastic pipes has been questioned by some researchers. Bernuth and Wilson [9] showed data from several sources indicating that the method did not work well for these pipes. The polyethylene pipes present a small roughness (*ε*≅8.116 *μ*m, [10]) and, given the practical limits of flow velocity adopted in the design of drip irrigation laterals, the smooth turbulent flow is predominant; hence, *f* depends only on the Reynolds number.

For the smooth turbulent flow, with 4000 ≤ *R*_*e*_ ≤ 10^5^, *f* is usually calculated by the Blasius equation or similar (equation (2)), specifically obtained for small-diameter polyethylene pipes [9, 11–13]:

(2) $$\begin{array}{c} f=\;\frac{a}{R_{e}^{b}}. \end{array}$$

In the Blasius equation, values of *a*=0.3164 and *b*=0.25 were determined when evaluating rigid smooth pipes. The factor *f* is dependent on pipe diameter, since it is a function of the Reynolds number:

(3) $$\begin{array}{c} R_{e}=\frac{4Q}{\pi \upsilon D}, \end{array}$$

where *υ* is the kinematic viscosity coefficient (m^2^·s^−1^), which is a function of the water temperature.

Bernuth and Wilson [9] showed that the Blasius equation presents suitable performance for small-diameter plastic pipes when *R*_*e*_ ≤ 100000. For noncollapsible polyethylene pipes with nominal diameters of 16, 20, and 25 mm and 2000 < *R*_*e*_ < 36000, Bagarello et al. [14] proposed a value of *a*=0.302. For nominal diameters of 12, 15, 18, 20, and 22 mm and 6000 < *R*_*e*_ < 72000, Frizzone et al. [13] proposed a value of *a*=0.300. For collapsible polyethylene pipes with wall thicknesses of 150, 200, and 250 *μ*m (6, 8, and 10 mil), results by [3] indicated that *a*=0.285. All the mentioned studies assumed that *b*=0.25.

Sousa and Dantas Neto [15] presented an adaptation of the Blasius equation coefficients for calculation of the friction factor for noncollapsible polyethylene pipes. They proposed that *a* and *b* are expressed by a power-law function of the pipe diameters, such that the values of *a* and *b* decrease with increasing diameter. In their model, when the diameter is varied between 13 and 16 mm, the value of *a* decreases from 0.3068 to 0.2923 (4.73%) and *b* decreases from 0.249 to 0.244 (2.01%). By this approach, the accuracy of predictions was improved.

Changes in the pipe internal diameter due to the operating pressure influence the head loss and the system hydraulic conditions. Melo et al. [4, 16] studied head loss in small-diameter thin-walled polyethylene pipes and showed that changes in the pipe diameter due to variations in the operating pressure affect the head loss and the maximum length of the irrigation system lateral lines.

Rettore Neto et al. [6] developed a procedure to estimate the head loss along elastic pipes, based on equation (1), considering the variation of the pipe cross section due to the operating pressure. The proposed equation allows estimation of variations in the pipe internal diameter as a function of the modulus of elasticity of the material, the wall thickness, and the operating pressure. However, the equation does not consider that collapsible pipes may not present a perfect circular flow section when operated under low pressures, and it may lead to inaccurate predictions.

According to [3], by using small-diameter collapsible polyethylene pipes, adequate predictions of *J* should consider variations in the effective diameter of the pipe with the operating pressure. However, incorrect values of pipe diameter combined with inaccurate values of the friction factor generate inaccurate estimates of head loss with negative consequences for the design of lateral lines.

Although the hydraulic design procedures of drip irrigation systems are well established, it is necessary to understand the effect of operating pressure on the diameter of thin-walled emitting pipes as well as the consequences related to the head loss. In the case of thin-walled drip tapes with continuous labyrinth, the friction factor and head loss may be increased by the presence of the continuous labyrinth inside the pipe. The objective of this work was to investigate the friction factor and the head loss of thin-walled drip tapes with continuous labyrinth operated within a range of operating pressures.

## 2. Materials and Methods

The work was carried out at the Laboratório de Ensaios de Material de Irrigação, Departamento de Engenharia de Biossistemas, Escola Superior de Agricultura Luiz de Queiroz, Universidade de São Paulo (Piracicaba, SP, Brazil). During the experiments, we evaluated two models of thin-walled drip tapes with continuous labyrinth manufactured by NaanDanJain® and Golden Tree®. Specifications given by the manufacturer are shown in Table 1.

The width and height of the continuous labyrinths for both models were measured with a digital caliper (resolution of 0.01 mm). The width and height were 7.90 and 0.07 mm, respectively, for the Turbo Tape and 4.60 and 1.08 mm for the Silver Drip.

An automated test bench was developed and validated by [17] for determination of head loss along pipes. The experimental procedures were performed in the laboratory using pipe segments 20 m in length, leveled on the ground, and having sealed orifices. The pipe samples were obtained randomly from rolls of Turbo Tape and Silver Drip models. The flow rate was adjusted by a Belimo® LRB24-3 proportional valve, with a 1/2″ flow section, installed downstream of the tested pipe segment. The flow rate was monitored by a Krohne® electromagnetic flowmeter, model IFC010D, with a resolution of 0.01 m^3^·h^−1^, measurement range of 0 to 4 m^3^·h^−1^, and uncertainty of 0.5% of full scale (F. S.). The pressure drop along the pipe segment was measured by a differential pressure transmitter, Novus® model NP800H, with a resolution of 0.01 kPa, measurement range of 1 to 100 kPa, and uncertainty of 0.075% F. S. The bench has an electronic circuit managed by a supervisory application which oversees the acquisition of sensor data and control the process.

Head loss equations were obtained for inlet pressures ranging from 40 to 100 kPa in steps of 20 kPa. For each inlet pressure and each pipe segment with sealed emitters, the head loss was measured under increasing and decreasing flow rates in increments of 0.2 m^3^·h^−1^. For each flow rate, 30 pairs of points of flow rate and head loss were recorded. Measurements were made for 20 flow rates, totaling 600 pairs of points for each sample at each inlet pressure. Two samples of each emitting-pipe model were evaluated.

During the experiments, the water temperature was monitored by a Zurich® temperature transmitter, model TZD 420, with a resolution of 0.1°C, a measurement range of 0 to 50°C, and uncertainty of 0.5°C. The mean water temperature during the tests was recorded for correction of head loss to the reference temperature of 23°C. The correction of the water density as a function of temperature was done by the equation proposed by [18]. The water coefficient of kinematic viscosity (*υ*, m^2^·s^−1^) as a function of water temperature (*T*, °C) was calculated according to [19] using a simple power-law relation.

(4) $$\begin{array}{c} \upsilon =0.000006177\;T^{-0.603}. \end{array}$$

The head loss obtained with water at the test temperature (*T*_test_) was corrected to the reference temperature (*T*_23_) using a multiplication factor *λ*:

(5) $$\begin{array}{c} \lambda =\frac{\upsilon_{T_{23}}}{\upsilon_{T_{\text{test}}}}. \end{array}$$

Considering a constant diameter for the leveled pipe, the relationship between flow rate and head loss was studied for each of the inlet pressures. The empirical equations of head loss were adjusted as a function of the flow rate, using a power-law model, in the following form:

(6) $$\begin{array}{c} J=\beta Q^{m} \end{array}$$

where *J* is the pipe head loss (m·m^−1^); *Q* is the flow rate (m^3^·s^−1^); *β* is a coefficient related to the studied pipe and adjusted based on the experimental data; and *m* is a coefficient that relies upon the flow regime. For fitting purposes, *m* was assumed to be 1.75 in order to obtain an equation resulting from the combination of the Darcy–Weisbach equation (equation (1)) with equation (2), assuming that *b*=0.25. Similar approaches were described in [3, 9, 13, 14].

Since the pipe diameter (*D*) increases with the pressure head (*H*) and head loss decreases with the increase in diameter, an empirical model for predicting head loss is proposed in

(7) $$\begin{array}{c} J=kQ^{m}H^{\alpha }, \end{array}$$

where *k* is an empirical coefficient obtained experimentally; *H* is the inlet pressure head (m); and *α* is a coefficient, less than zero, which expresses the effect of the pressure head on the pipe internal diameter. Again, for fitting purposes *m* was assumed to be 1.75.

A power-law model (equation (8)) was used to correlate the pipe internal diameter (*D*, m) with the pressure head (*H*, m). The coefficient values for the pipes studied at 23°C were obtained experimentally in [5]:

(8) $$\begin{array}{c} D=cH^{d}. \end{array}$$

For Turbo Tape, *c*=0.0156 and *d*=0.013 (*R*^2^=0.9968), while for Silver Drip *c*=0.0155 and *d*=0.007 (*R*^2^=0.9962).

For the determination of experimental values of *f*, for each pressure head, experimental values of *J* and the corresponding pipe internal diameters at 23°C were used, applying equation (1). The obtained values of *f* were related to *R*_*e*_^−0.25^ through a linear regression to obtain the parameter of equation (2), whose value corresponds to the angular coefficient of a linear function.

We also evaluated the possibility of calculating the head loss by equation (9), which is obtained by combining equations (1), (2), and (8):

(9) $$\begin{array}{c} J=\frac{8{\left(4\right)}^{-b}}{\pi^{\left(2-b\right)}}\left(\frac{a}{g}\right)\upsilon^{b}\frac{Q^{\left(2-b\right)}}{{\left(cH^{d}\right)}^{\left(5-b\right)}}. \end{array}$$

From the experimental dataset, 70% of the data were used to adjust the coefficients of equations (6), (7), and (9), while the remaining 30% of the data were used for validation and performance analysis of the equations.

The maximum lengths of irrigation laterals were calculated using the Turbo Tape and Silver Drip emitting pipes with a maximum pressure head variation of 10%, according to the methodology used in [4]. For comparison with other lengths, we used values of *a* found in this study and the most common values available in the literature.

The quality of the head loss predictions against the observed values was analyzed for a 1 : 1 line using the root-mean-square error (RMSE) and the cumulative frequency distribution of errors [20, 21]. The RMSE quantifies the dispersion between observed and estimated values and, ideally, its value tends to zero. Analysis of the cumulative frequency distribution of errors is also useful for estimating errors of prediction because it provides a distribution of the relative errors associated with the cumulative frequency.

## 3. Results and Discussion

After analyzing the head loss in the pipe with sealed orifices, for each pressure head, equations were obtained to calculate the head loss (*J*) as a function of the flow rate (*Q*) (Table 2). Head-loss equations were also adjusted independently of the pressure head and high values of the determination coefficient (*R*^2^) were obtained.

A small decrease in the coefficient *β* with increases in the pressure head is observed in the equations shown in Table 2. This indicates a weak dependence of the operating pressure on the internal diameter. This dependence is greater for the Turbo Tape which has a smaller wall thickness. For Silver Drip, the *β* coefficient was reduced from 289183.70 to 287183.70 (0.69%) while the pressure head (*H*) increased from 4 to 10 m (60%). In this range of values of pressure head, the diameter increased from 15.65 to 15.75 mm (0.64%). For the Turbo Tape, the *β* coefficient was reduced by 2.94% in the same range of values of pressure head, while the diameter increased from 15.88 to 16.07 mm (1.18%). Melo et al. [4] studied head loss in flexible laser-perforated polyethylene pipes with *D* = 28 mm and *ξ* = 200 *μ*m and found a 19.34% reduction in the head loss with a 50% increase in the operating pressure.

Using the experimental data of head loss (*J*, m·m^−1^) as a function of the flow rate (*Q*, m^3^·s^−1^) and pressure head (*H*, m), empirical equations of head loss were adjusted for the two models of emitting pipes studied, obtaining equation (10) for the Turbo Tape and equation (11) for Silver Drip:

(10) $$\begin{array}{c} J=297553.1Q^{1.75}H^{-0.02706},\;R^{2}=0.9976, \end{array}$$

(11) $$\begin{array}{c} J=289934.4Q^{1.75}H^{-0.00221},\;R^{2}=0.9994. \end{array}$$

**Figure 1:**
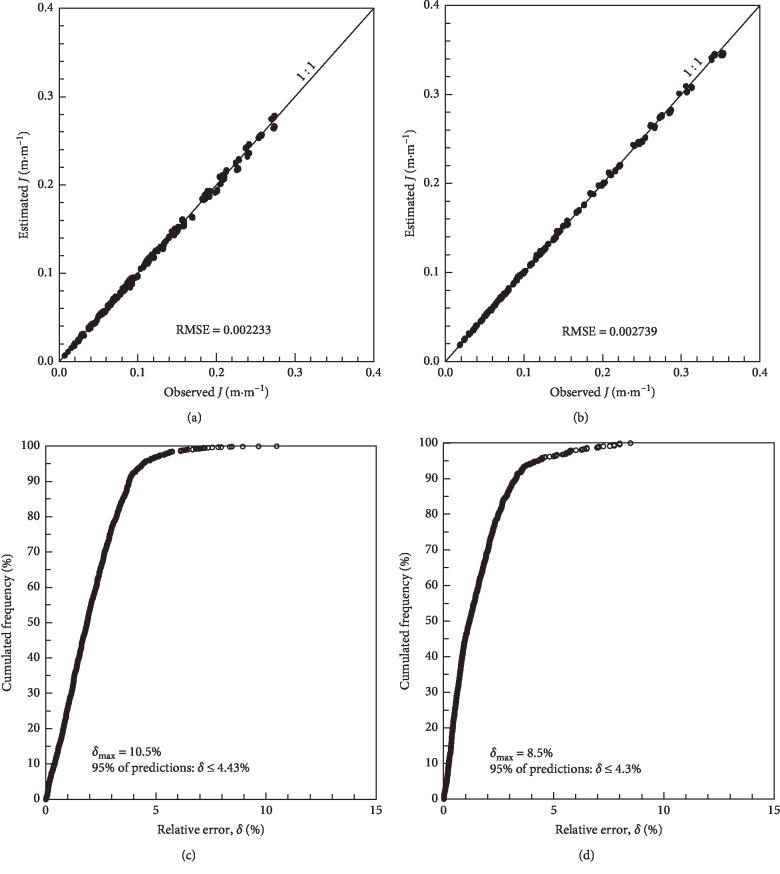
Concordance between observed and estimated head loss for the Turbo Tape (equation (10)) (a) and for the Silver Drip (equation (11)) (b); relative error by cumulative frequency for the Turbo Tape (equation (10)) (c) and Silver Drip (equation (11)) (d).

The agreement between the observed values of head loss and those estimated by equations (10) and (11) is presented in Figure 1. Adequate behavior is observed between the pairs of points around the 1 : 1 line. The RMSE is close to zero for both mathematical models. The pressure head has a small effect on the head loss because the values of the exponents of the pressure head are small, but this effect is greater for the Turbo Tape.

Predictions using equation (10) (Turbo Tape) presented a maximum relative error of 10.5%, with 95% of the estimates presenting a relative error of up to 4.43%. Predictions with equation (11) (Silver Drip) presented a maximum relative error of 8.5%, with 95% of the estimates presenting a relative error of up to 4.3%. This analysis indicates that equations (10) and (11) can be used to accurately calculate the head loss in the studied pipes.

Due to the weak dependence of *H* on *J*, empirical equations of head loss were fitted as a function of flow rate independently of the operating pressure head. High coefficients of determination (*R*^2^) were found for Turbo Tape (*R*^2^=0.9984, equation (12)) and Silver Drip (*R*^2^=0.9994, equation (13)):

(12) $$\begin{array}{c} J=281028.74Q^{1.75},\;4818\leq R_{e}\leq 32855, \end{array}$$

(13) $$\begin{array}{c} J=288849.08Q^{1.75},\;6276\leq R_{e}\leq 35626. \end{array}$$

Considering that the operating pressure of the Turbo Tape specified by the manufacturer is 80 kPa, the use of equation (12) instead of equation (10) results in a head-loss overestimation of 0.334%. For the Silver Drip, which has a larger wall thickness and expands less with the operating pressure, the use of equation (13) instead of equation (11) provides an overestimation of only 0.053%, demonstrating that its diameter is less sensitive to the operating pressure.

Provenzano et al. [3] affirm the pressure head along a drip irrigation lateral line which is influenced by the friction head loss and terrain elevation. In addition, the geometry of polyethylene collapsible pipes varies along the lateral length due to pressure decrease, which complicates calculation of head loss [6]. A mean diameter may be used as an approximation to compute accurately the friction head loss along a lateral [3, 22]. In the design of an irrigation lateral line, the maximum lateral length is calculated to obtain small variation in pressure head along the lateral (e.g., 5%), which is required to attain high Emission Uniformity (EU). For instance, supposing the lateral inlet pressure head is 8 m, then the minimum pressure head will be 7.6 m. Under such conditions, the diameter decrease along the whole lateral length would be 0.064% and 0.125% for the models Silver Drip and Turbo Tape, respectively. More accurate estimation of head loss might be obtained using a step-by-step procedure computing diameter values as a function of the pressure head along each segment of the lateral.

Thompson et al. [22] evaluated polyethylene collapsible pipes and they did not observe significant differences in friction head loss while comparing results using the hydraulic diameter and a mean diameter. Although the hydraulic diameter (four times the hydraulic radius) is generally used to calculate the friction head loss of noncircular conduits, Thompson et al. [22] report the hydraulic diameter underestimated the friction head loss and the use of a mean diameter results in results more accurate.

**Figure 2:**
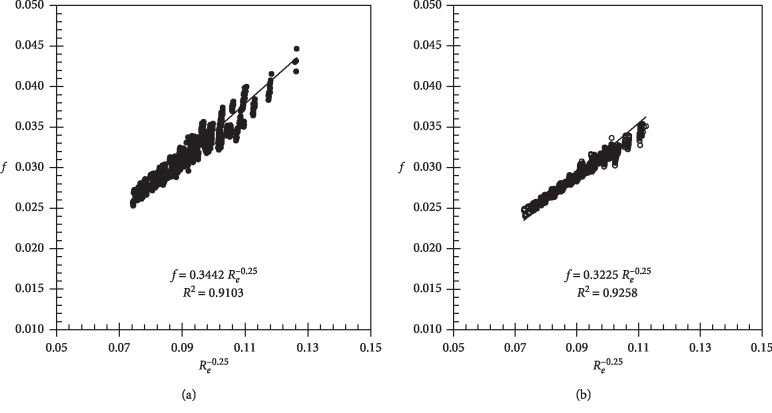
Friction factor (*f*) adjusted with *b*=0.25 for the Turbo Tape (a) and Silver Drip (b).

Figure 2 shows the curves of the friction factor (*f*) as a function of the Reynolds number (*R*_*e*_) for the two emitting pipes studied, using the experimental data, independently of operating pressure, with *b*=0.25. Assuming *f*(*R*_*e*_^−0.25^), the values of the coefficient *a* were 0.3442 and 0.3225 for the Turbo Tape and Silver Drip, respectively. Analyzing the modal value corresponding to the center of the highest frequency class, we found that *a*=0.3467 for the Turbo Tape and *a*=0.3233 for the Silver Drip. As these values converge to the values corresponding to the angular coefficients, it can be inferred that the probability distribution of the coefficients is close to the normal distribution.

In this research, the values of *a* were higher than those given by the Blasius equation for smooth pipes (*a*=0.3164) in [14] (*a*=0.302) and [13] (*a*=0.300) for noncollapsible polyethylene pipes and in [3] (*a*=0.285) for collapsible polyethylene pipes. Although the studied pipes are made of collapsible polyethylene, they have a continuous labyrinth welded into the pipe inner wall, causing an increase in the friction factor. The Turbo Tape presented a higher friction coefficient than the Silver Drip. A justification for this is the difference in the width of the continuous tape of emitters, since the Turbo Tape presents a greater width (7.9 mm) than the Silver Drip (4.6 mm). The approach proposed by [15] to obtain *a* and *b* relates these parameters to the pipe diameter. If we consider the operating pressure of 80 kPa, as recommended by the manufacturers, the diameters of the pipes determined hydraulically are 16.03 and 15.73 mm for Turbo Tape and Silver Drip, respectively. In this case, for the Turbo Tape, *a*=0.2918 and *b*=0.2440; for the Silver Drip, *a*=0.2931 and *b*=0.2444. These values are also lower than those obtained in this work.

The expanded measurement uncertainty of the flowmeter used during the experiments was 0.5% of the full scale. Since the full scale corresponds to 4 m^3^·h^−1^, the expanded uncertainty is 0.02 m^3^·h^−1^ with 95% coverage probability (normal distribution). For the Turbo Tape, which corresponds to the most critical case, the impact of flow rate uncertainty on friction losses may be estimated analyzing equation (12). For a flow rate of 1.00 ± 0.02 m^3^·h^−1^, the corresponding friction loss is 0.168 ± 0.006 m·m^−1^. Therefore, the measurement uncertainty in flow rate leads to about 3.5% of uncertainty in friction loss predictions, which may be assumed as a small uncertainty.

The parameters of equations (2) and (8) were applied in equation (9) to each pipe model to analyze the use of the Darcy–Weisbach equation to calculate the head loss in the studied pipes, with *b*=0.25 and *D* as a function of the pressure head. For the Turbo Tape, *a*=0.3442, *c*=0.0156, and *d*=0.013 (equation (14)). For Silver Drip pipe, *a*=0.3225, *c*=0.0155, and *d*=0.07 (equation (15)). These equations are expressions of the Darcy–Weisbach equation, rewritten with the experimental values of the coefficients specified for the temperature of 23°C (*υ* = 0.932515 × 10^−6^ m^2^·s^−1^).

(14) $$\begin{array}{c} J=8.3199\times 10^{-4}\frac{Q^{1.75}}{{\left(0.0156H^{0.013}\right)}^{4.75}}, \end{array}$$

(15) $$\begin{array}{c} J=7.7954\times 10^{-4}\frac{Q^{1.75}}{{\left(0.0155H^{0.007}\right)}^{4.75}}. \end{array}$$

**Figure 3:**
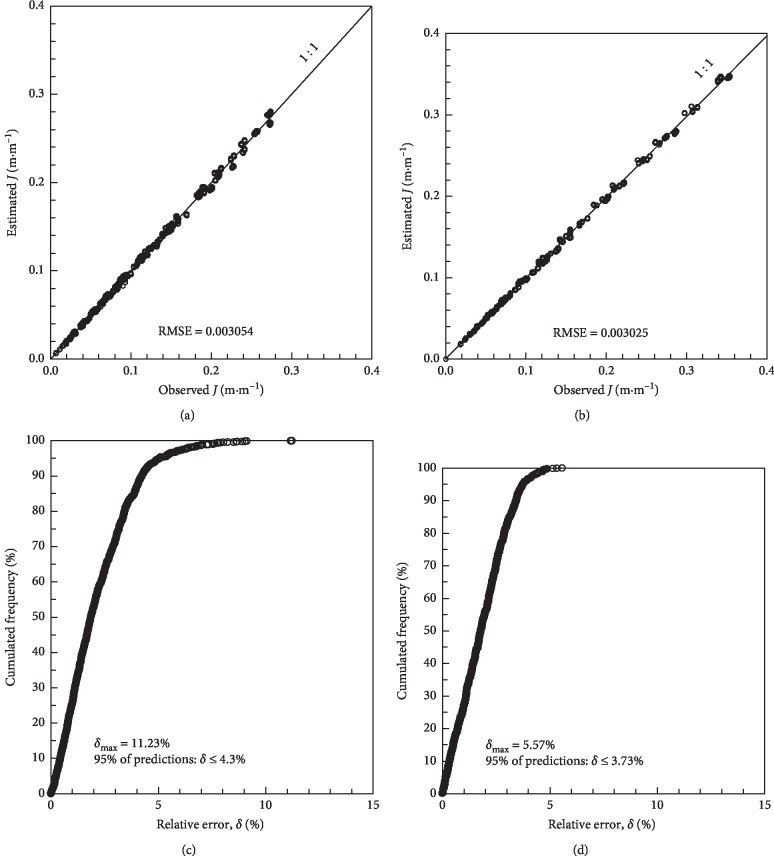
Concordance between observed and estimated head loss for the Turbo Tape (equation (14)) (a) and for the Silver Drip (equation (15)) (b); relative error according to cumulative frequency for the Turbo Tape (equation (14)) (c) and Silver Drip (equation (15)) (d).

Figure 3 shows the concordance between the estimated and observed head loss values (equations (14) and (15)). The RMSE is low, indicating a small mean deviation between the observed and predicted values. The cumulative frequency distribution of the relative errors indicated that for the Turbo Tape, the maximum error among the predictions is 11.23% and 95% of the estimates present an error of up to 4.3%. For the Silver Drip, the maximum error found was 5.57%, and 95% of the estimates presented relative errors of up to 3.73%.

**Figure 4:**
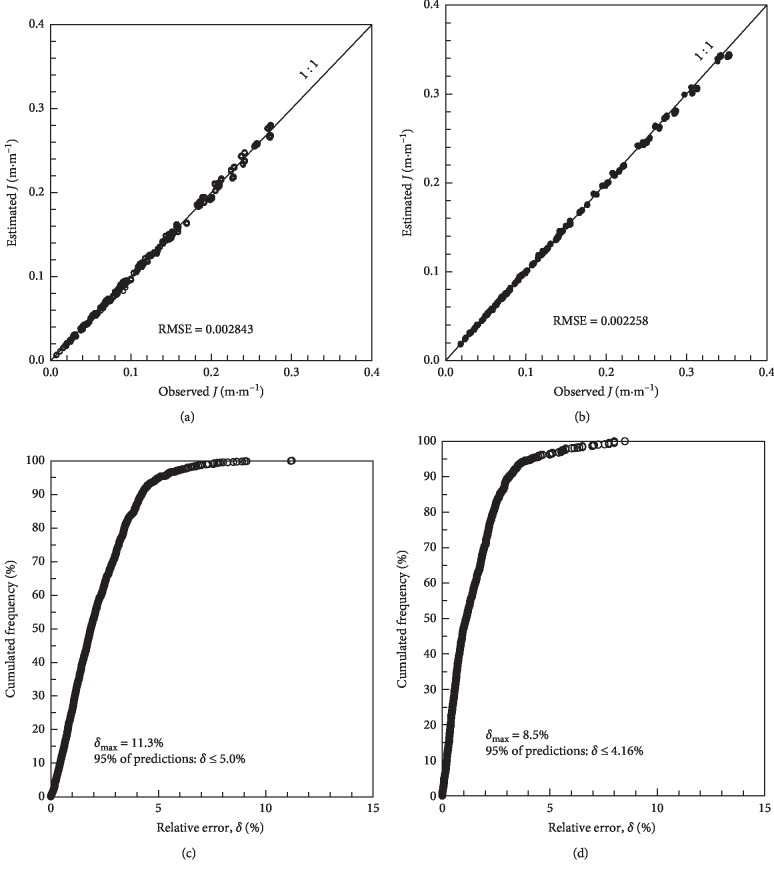
Concordance between observed and estimated head losses for the Turbo Tape (equation (14)) with a mean diameter of 16.01 mm (a) and for the Silver Drip (equation (15)) with mean diameter of 15.71 mm (b); relative error according to cumulative frequency for the Turbo Tape (c) and Silver Drip (d).

The performance of the Darcy–Weisbach equation was also analyzed when an optimum mean diameter, independent of the operating pressure head, was used in equations (14) and (15). In this case, the diameters that minimized RMSE were 16.01 mm for Turbo Tape and 15.71 mm for Silver Tape.  Figure 4 shows the agreement between the estimated and observed values of *J* for the two models of emitting pipes, for which good performance indices were found. For the Turbo Tape, the distribution of cumulated frequencies indicated a maximum error of 11.23%, and 95% of errors were equal to or less than 5%. For the Silver Drip, the maximum error was 8.5%, and 95% of the errors were up to 4.16%. It is proposed, therefore, that the head loss in the studied pipes be calculated by the Darcy–Weisbach equation using the mean diameters presented and the friction factor *f* with *b*=0.25 and *a*=0.3442 for the Turbo Tape and *a*=0.3225 for the Silver Drip, regardless of the operating pressure head within the limits analyzed in the study. It is observed that the diameter of 16.01 mm for the Turbo Tape corresponds to that obtained with *H* = 7.36 m, and for the Silver Drip, the diameter of 15.71 mm would be obtained for *H* = 6.84 m.

**Figure 5:**
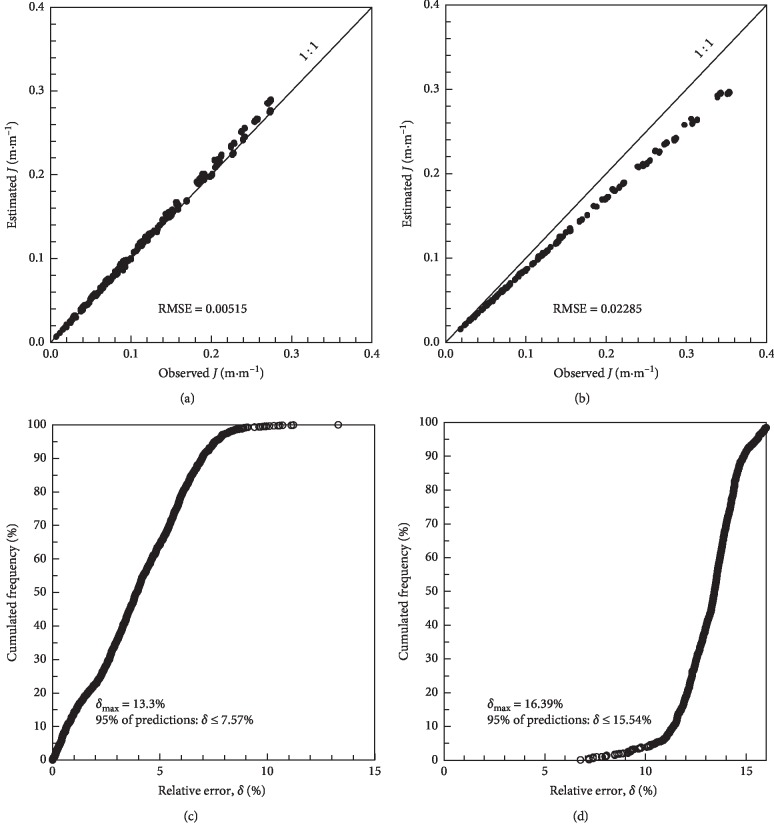
Concordance between observed and estimated head loss with the manufacturer's reported diameter for the Turbo Tape (equation (14)) with diameter of 15.9 mm (a) and for the Silver Drip (equation (15)) with mean diameter of 16.22 mm (b); relative error by cumulative frequency for the Turbo Tape (c) and Silver Drip (d).

The manufacturers of these pipes recommend an operating pressure of 80 kPa and report diameters of 15.90 mm for Turbo Tape and 16.22 mm for Silver Drip. In these cases, the error due to the calculation of head loss by the Darcy–Weisbach equation may be relevant (Figure 5). For the Silver Drip (Figure 5), the error was greater than that for the Turbo Tape. It is observed in Figure 5, especially for the Silver Drip, that the points are misaligned with respect to the straight line and the RMSE increased in relation to the one found for the optimal diameter. Turbo Tape presented a smaller deviation because the diameter reported by the manufacturer is close to the optimum diameter. For the Turbo Tape, the distribution of cumulated frequencies indicated a maximum error of 13.3%, and 95% of errors were equal to or less than 7.57%. For the Silver Drip model, the maximum error was 16.39%, and 95% of the errors were up to 15.54%.

Table 3 shows the comparison between the maximum lengths of lateral lines with Turbo Tape and Silver Drip, calculated with the values of *a* obtained in this research and some values available in the literature. The maximum lengths of the lateral lines were estimated, adopting the criterion of 10% of maximum variation of pressure in the lateral line under the inlet pressure head of 8 m. It was found that for *a*=0.285, as indicated by [3] for collapsible polyethylene pipes, the maximum length calculated for the lateral line with the Turbo Tape was 7.1% higher than the length calculated with *a*=0.3444, as indicated in this work, and 3.1% higher than that calculated with *a*=0.3164, as commonly referenced in the literature. The Silver Drip presented smaller differences in the calculation of the maximum length, because the value of *a* for this pipe is smaller than that for the Turbo Tape.

## 4. Conclusions

The experimental investigation indicated that the friction factor in the Darcy–Weisbach equation can be accurately described using a power-law model similar to the Blasius equation but characterized by coefficient *a*=0.3442 for the Turbo Tape and *a*=0.3225 for the Silver Tape. The values of this coefficient were higher than those reported in the literature for polyethylene pipes.

The influence of the pressure head on the diameter for the purpose of calculating the head loss can be neglected. The two approaches, considering the variation of the diameter with the pressure head and considering an optimum average diameter for the calculation of head loss by the Darcy–Weisbach equation, produce similar results, allowing accurate predictions of head loss. For both models of pipes, 95% of the head-loss estimates presented relative errors less than 5%.

For the Turbo Tape, the optimum diameter for calculating the head loss is 16.01 mm, which is very close to the value of 15.9 mm indicated by its manufacturer. For the Silver Drip model, the optimum diameter is 15.71 mm, while the manufacturer gives a value of 16.22 mm, which produces a relevant error in the head-loss calculation.

## Figures and Tables

**Table 1:** Specifications of the thin-walled drip tapes with continuous labyrinth evaluated during the experiments.

Manufacturer	Model	*D* ^(1)^ (mm)	*ξ* ^(2)^ (*μ*m)	*P* ^(3)^ (kPa)	*q* ^(4)^ (L·h^−1^)	*S* ^(5)^ (m)
NaanDanJain	Turbo tape	15.90	150	80	1.1	0.10
Golden tree	Silver drip	16.22	200	80	1.1	0.20

(1) Internal diameter; (2) wall thickness; (3) nominal pressure; (4) nominal flow rate; (5) orifice spacing.

**Table 2:** Equations of head loss (*J*, m·m^−1^) as a function of flow rate (*Q*, m^3^·s^−1^) for Turbo Tape (TT, *ξ* = 150 *μ*m) and Silver Drip (SD, *ξ* = 200 *μ*m).

Emitting pipe model	Test pressure (kPa)	*R*_*e*_ range	Equation	*R* ^2^
TT	40	4818 to 17383	*J* = 287455.85*Q*^1.75^	0.9984
TT	60	6454 to 23177	*J* = 282964.87*Q*^1.75^	0.9977
TT	80	6887 to 29092	*J* = 281889.13*Q*^1.75^	0.9991
TT	100	12741 to 32855	*J* = 279015.61*Q*^1.75^	0.9983
SD	40	6275 to 15337	*J* = 289183.70*Q*^1.75^	0.9993
SD	60	8947 to 22490	*J* = 288884.16*Q*^1.75^	0.9988
SD	80	11731 to 27427	*J* = 288696.89*Q*^1.75^	0.9997
SD	100	7954 to 31414	*J* = 287183.70*Q*^1.75^	0.9994

**Table 3:** Maximum lengths calculated for lateral lines with Turbo Tape (TT) and Silver Drip (SD) thin-walled drip tapes with continuous labyrinth for different values of *a* reported in the literature with *b*=0.25, using estimated mean diameters and Darcy–Weisbach's equation.

Pipe model	*D* (mm)	*a*	*L*_max_ (m)	Maximum length variation (%)
TT	16.01	0.285	69.5	+7.1
TT	16.01	0.3164	66.9	+3.1
TT	16.01	0.3442	64.9	—
SD	15.71	0.285	104.6	+4.7
SD	15.71	0.3164	100.6	+0.7
SD	15.71	0.3225	99.9	—

## Data Availability

The experimental data used to support the findings of this study are available from the corresponding author upon request.
